# Real-time visualization of epileptic seizures using photoacoustic imaging with a peroxynitrite-responsive manganese(ii) texaphyrin†

**DOI:** 10.1039/d5sc00568j

**Published:** 2025-03-18

**Authors:** Yaguang Ren, Calvin V. Chau, Tao Chen, Jingqin Chen, Yu Hu, Zhonghua Lu, James T. Brewster, Jonathan F. Arambula, Rongkang Gao, Adam C. Sedgwick, Jonathan L. Sessler, Chengbo Liu

**Affiliations:** a Research Center for Biomedical Optics and Molecular Imaging, Key Laboratory of Biomedical Imaging Science and System, Shenzhen Institutes of Advanced Technology, Chinese Academy of Sciences Shenzhen 518055 China cb.liu@siat.ac.cn; b Department of Chemistry, The University of Texas at Austin Austin Texas 78712-1224 USA; c Guangdong Provincial Key Laboratory of Brain Connectome and Behavior, The Brain Cognition and Brain Disease Institute, Shenzhen Institute of Advanced Technology, Chinese Academy of Sciences, Shenzhen-Hong Kong Institute of Brain Science Shenzhen 518055 China; d Department of Chemistry, King's College London 7 Trinity Street London SE1 1DB UK; e InnovoTEX Inc. 3800 N. Lamar Blvd Austin Texas 78756 USA; f School of Optics and Photonics, Beijing Institute of Technology Beijing 100089 China

## Abstract

Real-time visualization and tracking of epileptic seizures are important for studying epilepsy pathogenesis and treating epilepsy; however, the requisite sensing is extremely challenging, primarily due to the transient and intricate nature of neural activity associated with epilepsy. The onset of epilepsy is closely correlated with increases in peroxynitrite (ONOO^−^) levels, a reactive nitrogen species that can serve as a biomarker for epilepsy. However, the fleeting biological half-life and high reactivity of ONOO^−^ has historically impeded its direct visualization within the epileptic brain. This study explores the efficacy of manganese(ii) texaphyrin (MMn), a water-soluble and stable expanded porphyrin, in dynamically sensing ONOO^−^ and providing real-time tracking of epileptic seizures using a custom-built photoacoustic imaging (PAI) setup. UV-vis spectral analyses established the preferential sensitivity of MMn to ONOO^−^ over other reactive oxygen species (ROS), as well as its effectiveness through multiple usage cycles when rejuvenated *via* reaction with suitable reducing agents. This selectivity was recapitulated *in vitro* as determined through PAI experiments. *In vivo* application of this technique revealed that MMn administered intravenously crosses the blood–brain barrier (BBB) in a pentylenetetrazole (PTZ)-induced epilepsy mouse model and provides an observable 14.1 ± 3.7% reduction in photoacoustic (PA) signal intensity within the hippocampal region during epileptic seizures. Multiple decreasing–increasing cycles of PA signal intensity could be detected in the hippocampal region in this model; the observed effect thus mirrors closely the course of epileptic seizures inferred from mouse tail curling. Similar cyclical patterns were also seen in the motor cortex, a finding consistent with the extensive spread of epileptic activity throughout the brain. To the best of our knowledge, the present investigation represents the first real-time visualization and tracking of epileptic seizures using a peroxynitrite-specific sensing probe in combination with photoacoustic imaging (PAI). This approach enables deeper brain imaging while simultaneously capturing dynamic ONOO^−^ fluctuations, offering biochemical insights into epilepsy pathogenesis. By integrating deep-tissue imaging with neurochemical monitoring, this method lays the foundation for potential advances in epilepsy management and treatment.

## Abstract 2

Photoacoustic imaging as detailed in this report may allow the limitations associated with the real-time visualization and tracking of epileptic seizures to be overcome.

## Graphical abstract

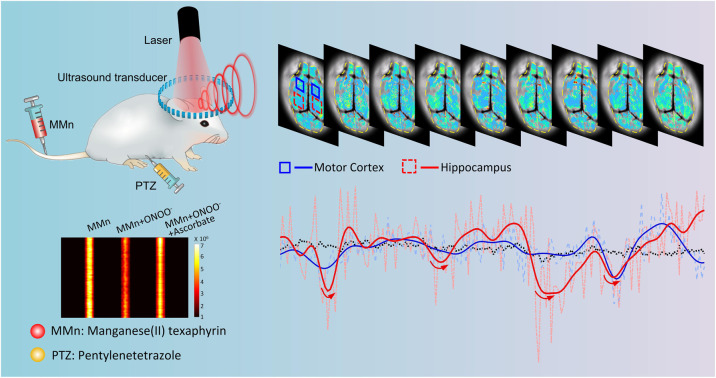

## Introduction

Epilepsy is a common neurodegenerative disorder characterized by recurrent epileptic seizures due to sudden, abnormal discharges of neurons in the brain.^1–3^ This disease can have serious adverse effects on both the physical and mental status of patients. The cause of epileptic seizures can be brain injury or genetic abnormalities, but for approximately half of epilepsy patients, the etiology is unknown.^4^ Epilepsy diagnoses are usually based on symptoms, physical signs, and changes in electroencephalogram (EEG) patterns, which collectively do not always localize the seizure onset zone accurately due to the inherent complexity of seizure semiology and propagation. Neuroimaging or structure-based imaging techniques, including computed tomography (CT) scans and magnetic resonance imaging (MRI),^5,6^ have advanced our capability to diagnose and manage epilepsy; nevertheless, there are inherent limitations to their efficacy and specificity, particularly in the real-time visualization and characterization of seizures. Given these challenges, increasing effort is being devoted to novel approaches in epilepsy monitoring that might improve the specificity and sensitivity of seizure detection and provide a greater understanding of the neurochemistry and pathophysiology of epilepsy.^7,8^

Previous studies have shown that epilepsy is closely associated with oxidative stress, in which numerous superoxide anion radicals (O_2_˙^−^) and nitric oxide radicals (NO˙) are continuously generated to form peroxynitrite (ONOO^−^).^9^ ONOO^−^ over-expression is not only considered a key causative factor for epilepsy but is also associated with the progression of epilepsy and can serve as a biomarker for epileptic seizures.^10^ Therefore, sensitive, specific visualization of ONOO^−^ is important for studying the neurochemical change of epileptic seizures. However, due to its high reactivity and short biological half-life, the real-time visualization of ONOO^−^*in vivo* has proved difficult.^11,12^ Specific fluorescent probes, such as rhodamine-based PF-6, coumarin-derived MitoPY1, and boronate-based HP green,^13–16^ have demonstrated potential in detecting ONOO^−^ in cellular models and animal brain tissues during epileptic events. These probes exploit the reactivity of ONOO^−^ to promote fluorescence modulation, thus serving as indicators of oxidative stress associated with seizure activity. Despite the promise these probes offer, their practical application is hampered by two significant limitations. Firstly, the imaging depth achieved with these fluorescent probes is typically restricted to superficial cortical layers due to the scattering and absorption of light in biological tissues. This limitation is particularly pertinent when attempting to monitor deeper brain loci that may be involved in the initiation and propagation of seizures. Secondly, many of the currently available fluorescent probes, such as PF-6 and MitoPY1, exhibit a response time on the order of minutes, which is sub-optimal given that seizure dynamics can evolve on a timescale of seconds. This temporal mismatch means that critical information regarding the onset and progression of seizure activity may not be captured accurately. As detailed below, we have now found that photoacoustic imaging (PAI), made possible by the use of a peroxynitrite responsive manganese(ii) texaphyrin (MMn), allows the real time monitoring of epileptic seizures in a mouse model.

Transition-metal complexes, particularly those involving expanded porphyrins, have attracted attention in the context of biomedical research, not only because of the sensing capacity they provide, but also for their therapeutic potential.^17–20^ Among the various expanded porphyrin complexes studied to date, MMn stands out due to several features that make it well-suited for applications in ONOO^−^ sensing and remediation within the brain.^21–23^ Firstly, it acts as a catalyst for the decomposition of ONOO^−^ into less-reactive nitrate and nitrite anions; this ability could potentially attenuate the oxidative stress associated with epileptic activity. The near-instantaneous response it displays helps the induced decomposition keep pace with the rapidity of neuronal hyperactivity associated with seizures. Secondly, the near-infrared (NIR) absorption of MMn, peaking at 725 nm in phosphate-buffered saline (PBS) at physiological pH, allows for deeper penetration of light into tissue.^24–26^ This characteristic is essential for imaging beyond the superficial layers of the brain, where light scattering and absorption significantly limit the effectiveness of traditional fluorescent probes.^27^ Furthermore, unlike many contrast agents that are limited to a single use due to irreversible reactions with ONOO^−^, MMn is capable of multiple turnovers. In addition to offering a potentially sustainable approach to ONOO^−^ imaging, this repeated usability could prove beneficial in studying chronic conditions like epilepsy, where monitoring over extended periods is often necessary.

**Fig. 1:**
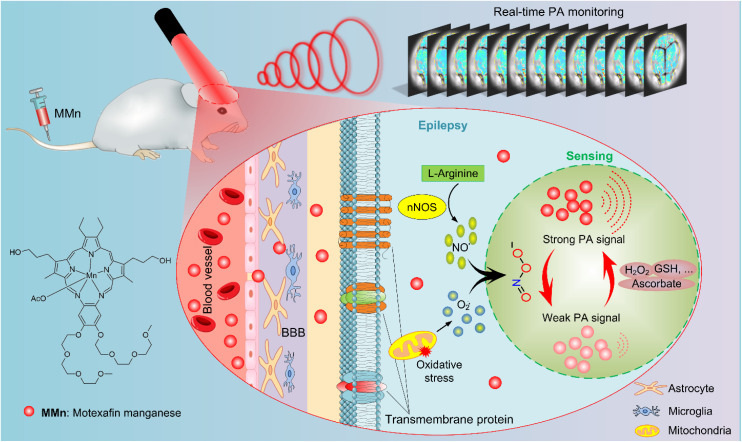
Schematic illustration of the mechanism proposed to underlie the real-time photoacoustic imaging (PAI) of peroxynitrite (ONOO^−^) in epileptic seizures using manganese(ii) texaphyrin (MMn). This diagram delineates the molecular processes thought to occur during epileptic seizures and the subsequent application of PAI for monitoring these events. In epileptic brains, the blood–brain barrier (BBB) is often compromised due to a confluence of factors, including inflammatory responses and oxidative stress triggered by seizure activity. A key event during seizures is the over-production of ONOO^−^, a compound formed by the rapid interaction of superoxide anion (O_2_˙^−^) and nitric oxide (NO˙). Following administration through tail vein injection, MMn effectively penetrates the disrupted BBB and interacts selectively and reversibly with ONOO^−^. This results in readily discernible differences in the PA signal intensity thus allowing seizure dynamics to be monitored directly in real time.

The real-time, non-invasive monitoring of ONOO^−^ levels beyond the surface strata of the brain requires imaging techniques with fast dynamics, a large field-of-view, and acceptable deep tissue imaging capabilities. PAI is a promising tool in this regard since it allows dynamic processes to be visualized with remarkable clarity and depth.^28,29^ PAI typically relies on a nanosecond pulsed laser to excite an exogenous contrast agent that absorbs the light energy and converts it into heat. The resulting local heating leads to transient thermoelastic expansion and the generation of detectable ultrasound waves.^30,31^ By integrating MMn as a peroxynitrite-sensitive contrast agent with PAI, we postulated that it might be possible to achieve the real-time visualization and understanding of epileptic seizures by monitoring dynamic changes in ONOO^−^ levels. This study was undertaken as a test of this proposition. We thus sought to assess the ability of MMn to penetrate the compromised blood–brain barrier (BBB) characteristic of seizures, interact with ONOO^−^, and cause a measurable decrease in the photoacoustic (PA) signal intensity. As discussed below, using a pentylenetetrazole (PTZ)-induced kindling mouse model, as well as MMn in conjunction with PAI, we were able to monitor in a continuous, real-time manner ONOO^−^ production during epileptic seizures, as evidenced by changes in PA signal intensity that are correlated with seizure activity in mice (Fig. 1).

## Results and discussion

### UV-vis spectral study on the interaction between MMn and ONOO^−^

**Fig. 2:**
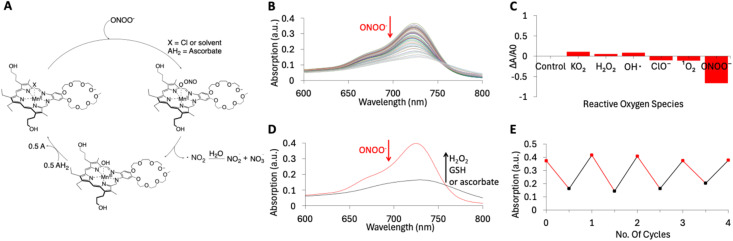
Comprehensive UV-vis spectroscopic analysis of the interaction between MMn and ONOO^−^. (A) Schematic view of the molecular mechanism underlying the reaction between MMn and ONOO^−^ expected to pertain in a biological context. (B) UV-vis spectral changes observed as MMn, dissolved in PBS (pH = 7.4) at a concentration of 20 μM, was titrated with increasing concentrations of ONOO^−^ (ranging from 0 to 30 μM) in PBS (pH = 7.4). (C) Reactivity of MMn towards ONOO^−^ relative to other reactive oxygen species (ROS) as inferred from UV-vis spectral studies in PBS (pH = 7.4). (D) UV-vis absorption spectrum of MMn (20 μM) recorded in the presence of ONOO^−^ (30 μM) and various reducing agents (H_2_O_2_, GSH, or ascorbate) (excess) in PBS (pH = 7.4). (E) UV-vis absorption intensity at 725 nm for MMn samples treated with ONOO^−^ and ascorbate across multiple cycles.

Mirroring earlier studies,^21^ in our hands, MMn proved capable of decomposing ONOO^−^ selectively over other reactive oxygen species (ROS) in a physiologically relevant environment, specifically in PBS buffer at pH 7.4. The proposed mechanism of interaction between MMn and ONOO^−^ is shown in Fig. 2A. Further mechanistic details are provided in the ESI text.† It is important to appreciate that MMn acts as a catalyst for ONOO^−^ decomposition. This inference is supported by the finding that upon titrating ONOO^−^ in concentrations ranging from 0 to 30 μM into solutions of MMn at 20 μM, the absorbance intensity at 725 nm ascribed to the MMn chromophore declines by approximately 60.0%; this is consistent with a robust reaction between MMn and ONOO^−^ (Fig. 2B). In contrast, other ROS such as hypochlorite (ClO^−^) and singlet oxygen (^1^O_2_) produced a decrease of less than *ca.* 10% under otherwise identical conditions (Fig. 2C). The introduction of common reducing agents, including hydrogen peroxide (H_2_O_2_), glutathione (GSH), and ascorbate, led to regeneration of the MMn absorbance intensity, allowing for subsequent interactions with ONOO^−^ (Fig. 2D). The efficiency of MMn to decompose ONOO^−^ was assessed over multiple rounds (Fig. 2E). While a slight reduction in effectiveness was noted over successive cycles, MMn exhibited a sustained capacity to catalyze the decomposition of ONOO^−^. This regenerative capability led us to consider that MMn may have a role to play as a reusable agent for detecting ONOO^−^ in biological contexts.

### *In vitro* and *in situ* study of PA behavior of MMn responsiveness to ONOO^−^

**Fig. 3:**
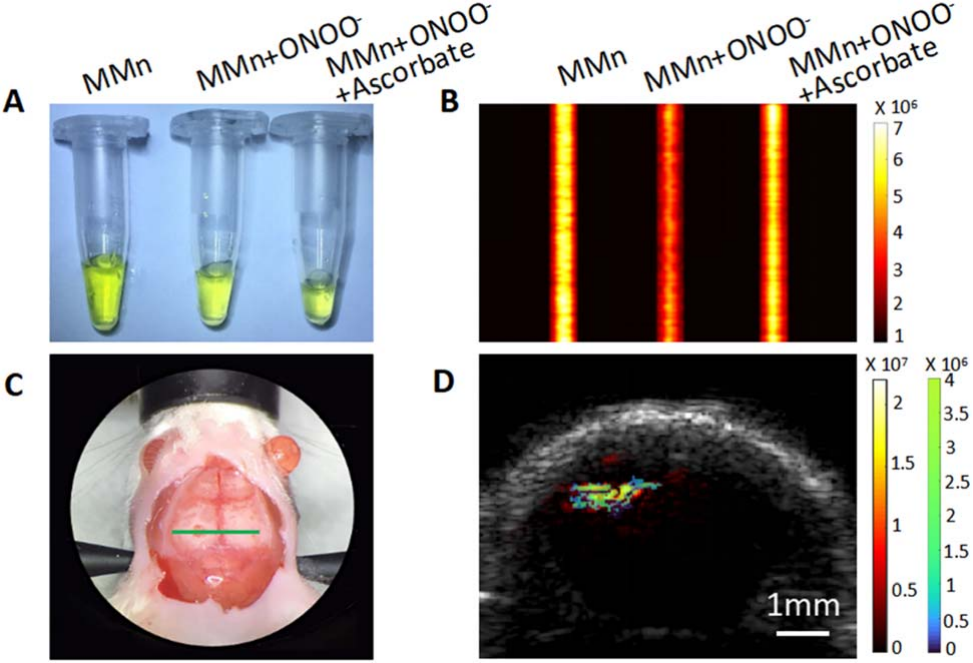
PA monitoring of the interaction of MMn with ONOO^−^ in both *in vitro* and *in vivo* settings. (A) Images of the test solutions used in the *in vitro* PAI studies: MMn dissolved in PBS (pH = 7.4) (20 μM, left), MMn with the addition of 30 μM ONOO^−^ (middle), and MMn with 30 μM ONOO^−^ and 50 μM ascorbate (right). (B) PA intensities of the test solutions. (C) Photograph of skull surface of a mouse. The green line marks the coronal plane corresponding to the hippocampus, as referenced in Fig. S2,† which was used for the PAI studies employing a linear-array system. (D) *In situ* PA imaging of a mouse brain following MMn injection. The hot color bar, represented by shades of red and yellow, represents the PA signal of MMn prior to seizure induction. In contrast, the cold color bar, shown in shades of blue and green, delineates the alterations in the PA signal after pentylenetetrazole (PTZ)-induced seizures. Together, the color coding is designed to provide a visual representation of the changes in brain chemistry due to seizure activity.

To study the putative selective interaction of MMn with ONOO^−^ under conditions of PA monitoring, within a PAI framework, MMn dissolved in PBS buffer (pH = 7.4), at a concentration of 20 μM was placed in slender plastic capillaries of 1.1 mm diameter and subjected to laser excitation (*cf.*Fig. 3A). A laser, tuned to emit at 725 nm (the lowest energy absorption maximum of MMn) with a fluence of 15 mJ cm^−2^, was used to photoexcite the sample. The PA signals were systematically recorded 250 times using a linear-array PAI system to remove the variability in laser pulse excitation. This procedure yielded maximum amplitude projection (MAP) images, as depicted in Fig. 3B (refer to Fig. S1† for a visual representation). Notably, in the presence of ONOO^−^ at a concentration of 30 μM, the mean intensity of PA signal attributed to MMn exhibited a statistically significant reduction of 70.6 ± 3.3%. The deviation, measured as the standard error of the mean (SEM), primarily results from fluctuations in the laser pulse excitation. This reduction was taken as evidence of an effective interaction between MMn and ONOO^−^, in accord with what was inferred on the basis of the UV-vis spectral studies discussed above. The introduction of ascorbate, a common reducing agent, at a concentration of 50 μM, resulted in a restitution of the PA signal intensity to 92.9% (SEM = 3.1%) of its initial value as shown in Fig. 3B. These findings thus provide support the suggestions that MMn can act as a catalytic probe for the *in situ* detection and quantification of ONOO^−^ and that the effects can be tacked in quantifiable terms using PAI. The dynamic nature of the response was thought to augur well for the use of MMn as an ONOO^−^ probe in an epilepsy mouse model where real-time imaging was considered imperative.

The next step in our study involved testing whether MMn would allow for the tracking of ONOO^−^*in vivo* and permit monitoring of the dynamics associated with epileptic seizures. The transition to *in vivo* experimentation was facilitated by the use of the same linear-array PAI system as used for the *in vitro* studies. Using an *n* = 5 and the chronic epileptic mouse model, MMn was administered into the hippocampal region *in situ* at a concentration of 400 μM. Prior to intervention, mice were anesthetized using isoflurane to achieve immobilization and mitigate distress. The scalp was surgically excised to reveal the cranial surface. A precise craniotomy with a diameter of approximately 2 mm was then executed directly overlying the hippocampal area. An infusion rate of 1 μL min^−1^ was meticulously maintained to administer a total volume of 3 μL. This protocol was designed to achieve uniform distribution throughout the target region, as outlined in the “*In situ* linear array PAI protocol” within the ESI Materials and methods section.† Following MMn administration and cessation of isoflurane anesthesia, seizures were pharmacologically induced with PTZ.

Given the complex vasculature of the brain and the strong light absorption by blood, a normalization procedure was used to discern the PA signals that could be ascribed to MMn. This normalization was first validated through dual-wavelength PAI experiments conducted *in vitro*, where the PA signals of MMn and blood were both evaluated at 725 nm and 800 nm (Fig. S3†). MMn exhibits minimal absorption at 800 nm, rendering its PA signal at this wavelength effectively negligible. This characteristic allowed us to attribute the PA signal at 800 nm predominantly to blood. Utilizing the known absorption coefficients of blood at the two wavelengths, we then calculated the blood-based contribution to the PA signal at 725 nm and extracted the PA signal specific to MMn at this wavelength (see ESI text†).

Our *in vivo* findings, as depicted in Fig. S4,† revealed a measurable decrease in the MMn PA signal at the injection site following PTZ induced seizures across three mice. On average, there was a 15.3 ± 2.0% decrease in signal intensity where the error corresponds to the SEM after seizure induction when compared to baseline measurements. The signal attenuation seen for this *in situ* administration portion of the study is attributed to the interaction of MMn with ONOO^−^ in the hippocampus during seizure activity. A visual comparison is presented in Fig. 3D; it employs a colorimetric scale where warmer tones represent the PA signal of MMn before seizure induction, and cooler tones depict the post-seizure signal variances. These preliminary *in situ* assessments lead us to suggest that MMn shows promise as an effective probe for the real-time PA tracking of ONOO^−^ fluctuations during seizures.

### Linear-array PAI of neurochemical changes deep in the brain upon intravenous MMn injection

As the next step in evaluating the efficacy of MMn as a contrast agent for detecting epileptic seizures within the brain, we tested its ability to permeate the BBB following tail vein injection. For this portion of the study, we established a chronic epilepsy model through repeated intraperitoneal injection of PTZ in male BALB/c mice as detailed in the “Animal models of epilepsy” portion in our Materials and methods section. Using this model, the PA signal in the hippocampus was monitored for 60 minutes post-intravenous MMn injection (1000 mM, 150 μL in saline) (Fig. S5†). An immediate and marked enhancement in the PA signal in the epileptic seizure mouse group was seen post-MMn injection, with an overall increase in PA intensity (12.3%; SEM = 2.4%) being seen in the epileptic seizure mouse groups, whereas no discernible change in the PA signal was seen in the saline epileptic seizure control group. On this basis, we suggest that MMn successful crosses the BBB and accumulates within the brains of epileptic mice.

In a control experiment involving normal mice, a rapid increase in the PA signal intensity was observed following MMn injection, mirroring the response in the epileptic seizure group. However, in contrast to what was seen in the epileptic models, the PA signal in normal mice quickly reverted to baseline post-injection (Fig. S5†). While not a proof, such a finding provides support for the expectation that MMn is unable to cross effectively the intact BBB present in healthy mice. It is thus subject to subsequent metabolic clearance through the scalp vasculature system.

Further support for the suggestion that MMn can pass the BBB in epileptic mice came from scanning electron microscopy tests involving brain slices (Fig. S6†). In this analysis, MMn was not detected in the brains of normal mice post-intravenous administration. In contrast, in epileptic mice, MMn was clearly visible within the brain slices (Fig. S6C†), confirming its presence. This observation aligns with the known disruption of the BBB in such models. Additionally, as a confirmatory measure, Evans blue dye, which is recognized as a permeant of BBB under compromised conditions, was observed within the brains of mice exhibiting epileptic seizures. This multi-modal approach provides confidence that the observed PA signal changes are attributable to the presence of MMn within the brain following seizure-induced BBB disruption.

**Fig. 4:**
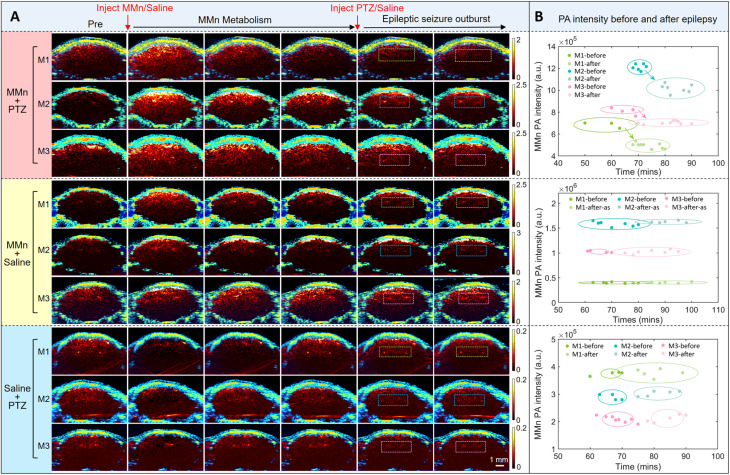
In-depth analysis of epileptic brain activity *via* linear-array PAI in a mouse model (*n* = 3). (A) The coronal views of mouse brains captured using the linear-array PAI system at specific time points: Before and after intravenous administration of MMn or saline, as well as prior and subsequent to intraperitoneal injection of either pentylenetetrazole (PTZ) to induce epileptic seizures or saline as a control. (B) Comparison of PA signal intensities across three distinct experimental groups: MMn + PTZ, MMn + saline, and saline + PTZ. Regions of focus are demarcated by dashed boxes. The 95% confidence-level error ellipses for each group are also shown. M1: mouse #1; M2: mouse #2; M3: mouse #3.

After establishing that MMn can penetrate the BBB in epileptic mice, we focused on assessing its effectiveness as a probe for detecting endogenous ONOO^−^ within the brain. Toward this end, we used the same linear-array PAI system as above to capture coronal views of PTZ-induced epileptic brains at different time points. The imaging cross-section is shown in Fig. S2† and key PA images are provided in Fig. 4A. In the MMn + PTZ experimental group (*n* = 3), depicted in the upper subpanel of Fig. 4A, intravenous injection of MMn (1000 mM, 150 μL in saline) was followed by the removal of anesthesia. Prior to injecting with PTZ, the PA signal in the hippocampal region was monitored for 60 minutes. This was done to confirm that the MMn-derived signal was stable, thus ruling out metabolic variations in MMn levels as a possible source of PA signal fluctuations. Following MMn injection, the PA signal intensity increased initially. It then gradually declined, and eventually stabilized, a variation ascribed to baseline metabolism of MMn. After intraperitoneal PTZ injection, brain monitoring was continued for about 30 minutes with a focus on the hippocampal region (highlighted by dashed boxes). A comparison of images taken before and immediately after the onset of epileptic seizures revealed that, in all three mice making up the MMn + PTZ group, the hippocampal region was subject to a discernible signal decrease post-seizure. No epileptic seizures and consequently no signal decreases were observed in a control group that were treated with saline post-MMn injection (middle subpanel of Fig. 4A; MMn + saline; *n* = 3). Replacing MMn with saline prior to PTZ injection (lower subpanel Fig. 4A; saline + PTZ group; *n* = 3) likewise engendered no noticeable decrease in the PA signal post-seizure.

A detailed statistical analysis on the average intensities of the PA signals in the hippocampal regions demarcated by dashed boxes in Fig. 4A was performed. The 95% confidence-level error ellipses for the PA signal intensity of each mouse, before and after epileptic seizure induction, are shown in Fig. 4B. Notably, the MMn + PTZ group exhibited an average decrease in the MMn PA signal intensity of 14.1% (SEM = 3.7%) after the onset of epileptic seizures. This contrasts with the control groups (saline + PTZ and MMn + saline), where no significant PA signal intensity changes were observed. Taken in aggregate, these findings lead us to suggest that MMn, upon crossing the disrupted BBB in epileptic models, interacts with ONOO^−^ generated during seizures, which in turn leads to the observed decrease in the PA signal intensity. This inference is further substantiated by the lack of similar PA signal intensity changes in the MMn + saline group, where epileptic seizures did not occur, or the saline + PTZ group, where seizures occurred in the absence of MMn administration and the PA signal ascribed to its presence.

### Circular-array PAI of neurochemical changes in multiple brain regions upon intravenous MMn injection

**Fig. 5:**
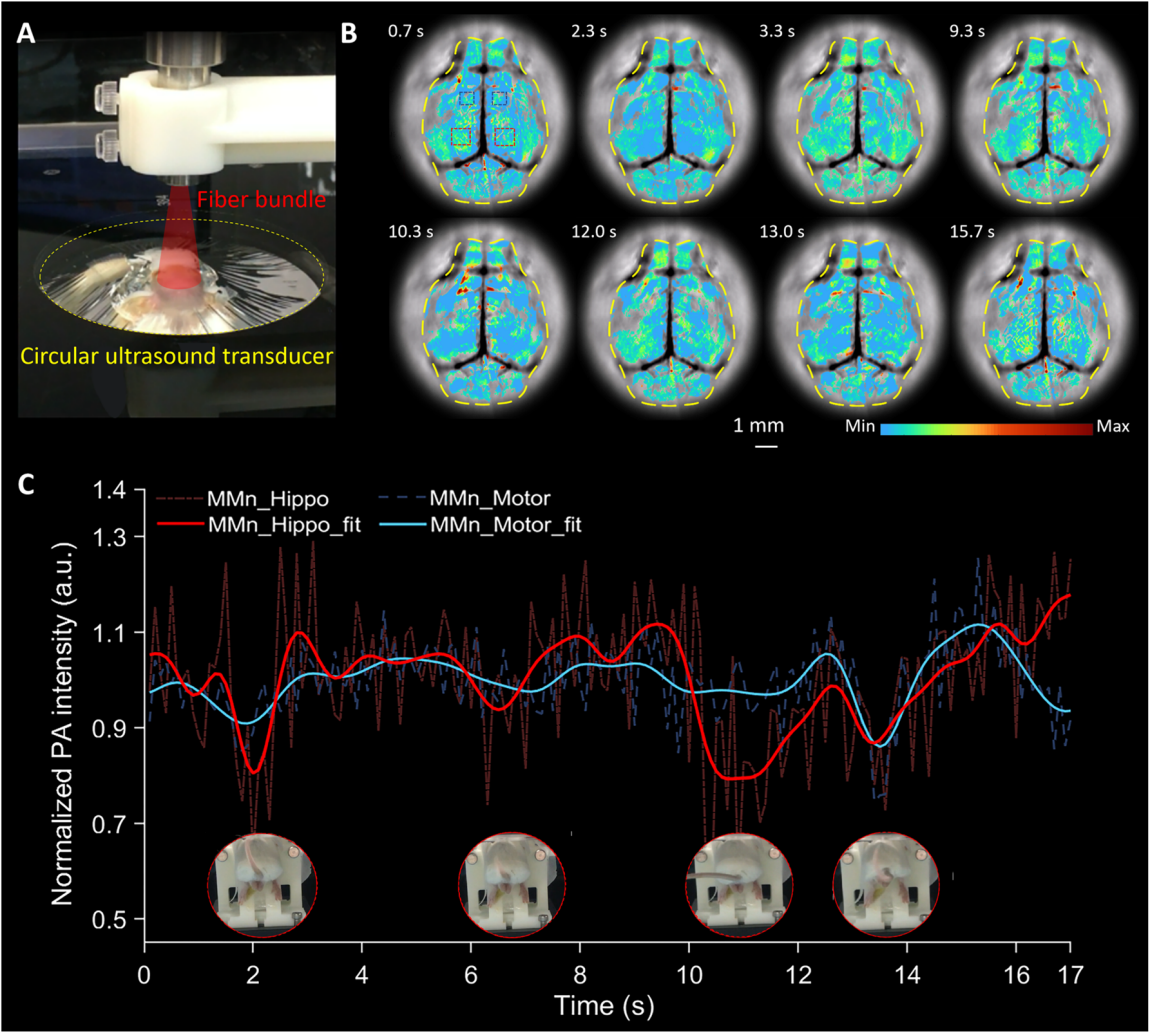
Real-time PAI of the mouse brain using a circular-array ultrasound transducer. (A) Photograph of the brain imaging setup with a circular-array ultrasound transducer. (B) Transverse view of multiple representative images selected from a 17 second segment of the recorded data. (C) Average MMn signal in two critical regions: the hippocampal region (indicated by two red dashed boxes in (B)) and the motor cortex region (marked by two blue dashed boxes in (B)). A low-pass filter based on the fast Fourier transform was applied to analyze the underlying trends in the signal, resulting in the fit lines shown by the red and blue curves. This allows for a comparative analysis of fluctuating MMn signals across different brain areas within the same 17 second data segment.

Brain activation during epileptic events can be rapid and dynamic, particularly foci initiation and propagation. We were thus keen to see if PA imaging using MMn would allow us to detect ONOO^−^ across different brain regions in our mouse model during a PTZ-induced epileptic seizure. For these studies, a custom-designed circular-array ultrasound transducer-based PAI system (Fig. 5A and S1†) was used that allowed the PA intensity in multiple brain regions to be mapped simultaneously. This setup, featuring a high imaging frame rate (20 frames per s), a large field-of-view, and a high acoustic central frequency (10 MHz), was specifically tailored to capture real-time changes in the MMn signal across the transverse cross-section of the mouse brain.

PTZ-induced seizures typically originate in the hippocampus and then spread throughout the brain, accompanied by an overproduction of ONOO^−^.^32^ Several representative images selected from a 17 second data segment, encapsulating multiple seizure events in a mouse, are displayed in Fig. 5B. The intensity of the MMn signal changes over time in different brain regions, such as those indicated by the red and blue boxes. We specifically analyzed the hippocampal region, marked by red boxes in Fig. 5B and the red region in Fig. S7,† depicting the average intensity of the MMn-derived PA signals as a red dashed curve in Fig. 5C. To elucidate the underlying trends in the signal, we employed a low-pass filter based on the fast Fourier transform, effectively minimizing extraneous disturbances. The filtered results are depicted as a solid red curve. A cyclical pattern of decreasing and increasing MMn signal intensity was observed, correlating with the onset of epileptic seizures, as evidenced by mouse tail curling (Fig. 5C and Movie S1†). These patterns were replicated in another mouse experiencing seizures (Fig. S8 and Movie S1, mouse #2†). They are considered to reflect a dynamic interaction between MMn and ONOO^−^, as well as the recovery of MMn facilitated by reducing agents like ascorbate. These processes are likely influenced further by brain and cellular functions.^33,34^ These cyclical signal changes were absent before PTZ administration (Fig. S9 and Movie S2†) and began to diminish post-seizure (Fig. S10 and Movie S3†). This attenuation of signal cycling may reflect the finite quantity of ONOO^−^ or the loss of MMn within the complex physiological milieu post-seizure.

Extending our analysis beyond the hippocampus, we quantified MMn signals in the motor cortex region, as indicated by blue boxes in Fig. 5B and blue regions in Fig. S8.† We recorded the average intensity and filtered signal changes, represented by the blue dashed and solid curves, respectively, in Fig. 5C. At specific time points, including at 2 s, 7 s, and 13.5 s, we observed fluctuations in the motor cortex region that mirrored those documented in the hippocampal region (red curves in Fig. 5C and Movie S1†). This parallel trend in both the blue and red curves suggests a widespread propagation of epileptic activity across these brain regions. The consistent cyclical changes in MMn signal intensity in response to seizure events, observable in both regions, were absent before PTZ administration and gradually diminished post-seizure.

## Discussion

In this study, we introduce a combined probe- and PA imaging-based approach that allows the real-time monitoring of ONOO^−^, a critical marker closely linked to neurochemical changes during epileptic seizures. A key feature is the use of MMn, a small molecule with the ability to cross the BBB of the epileptic brain that reacts selectively with ONOO^−^. By integrating MMn with PAI, it proved possible to observe a statistically significant reduction in PA signal intensity within the hippocampal region during epileptic seizures in a mouse model. This was coupled with the detection of distinct decreasing–increasing cycles of PA signal intensity in this area, which proved closely correlated with the phases of epileptic seizures, as evidenced by characteristic tail curling in mice.

The results reported here underscore the feasibility of using MMn as a reusable probe in the PA imaging of ONOO^−^. Our study primarily focuses on dynamic, real-time monitoring, future work involving the use of biochemical techniques such as ICP-MS or LC-MS could further validate the biodistribution and pharmacokinetics of MMn, could provide insights into its accumulation, clearance, and long-term stability in brain tissues. Likewise, ONOO^−^ scavengers or ROS inhibitors could, in theory, could help validate the selectivity of MMn *in vivo*; however, their use in chronic epilepsy models presents significant challenges due to potential physiological alterations that may complicate data interpretation.

Beyond validating the specificity of MMn, this study underscores the broader role of PAI in assessing epilepsy-related neurochemical processes and evaluating antioxidative treatments. A major challenge in extending this technique to continuous seizure monitoring lies in the large data volume and the computational demands associated with PAI acquisition and processing. However, advances in AI-driven signal analysis offer promising avenues for overcoming these limitations, potentially enabling long-term, real-time tracking of seizure-related neurochemical dynamics. Advances in both probe design and imaging instrumentation could also allow for deeper insights into seizure-related neurochemical activity. In particular, expanding PAI- from two-dimensional cross-sectional imaging to three-dimensional (3D) volumetric imaging would allow a more comprehensive understanding of seizure onset, propagation, and neurochemical changes across the entire brain. Current efforts are focused on addressing these challenges, paving the way for more advanced applications of PAI in epilepsy research.

## Experimental

### Synthesis and characterization of MMn

MMn was synthesized following established protocols.^35,36^ For purification, MMn was processed using reversed-phase tC18 solid-phase extraction (SPE) columns (Waters Sep-Pak, 10 g). The elution was performed with a gradient of acetonitrile (10% to 35%) in either a 0.1 M ammonium acetate or a 0.1% acetic acid aqueous solution. Its purity was confirmed through reverse-phase high-performance liquid chromatography (RP-HPLC), aligning with prior analyses.^25^ RP-HPLC was conducted using a Thermo Scientific Dionex Ultimate 3000 HPLC system, equipped with a photodiode array (PDA) detector. Separation was achieved on a Syncronis C18 column (5 μm, 4.6 × 250 mm, Thermo Scientific). The mobile phase comprised a gradient of acetonitrile (10% to 99% over 20 minutes) in water, both containing 0.1% acetic acid, at a flow rate of 1.2 mL min^−1^. MMn detection was conducted at wavelengths of 254, 470, and 740 nm. UV-vis spectra were obtained with a Varian Cary 5000 spectrophotometer at ambient temperature. A 10 mm path length quartz cuvette was used for all measurements.

### *In vitro* synthesis and characterization of ONOO^−^

Four separate solutions (10 mL) of NaNO_2_ (0.6 M), HCl (0.68 M) with H_2_O_2_ (0.72 M), and NaOH (3.6 M) were prepared and stored in the freezer at −20 °C for 30 minutes. Then, the solutions were transferred to an ice bath and allowed to sit for 30 minutes. The NaNO_2_ solution was added to a 100 mL beaker and placed in an ice bath with >500 rpm stirring. The HCl/H_2_O_2_ solution was added to the NaNO_2_ solution, followed by immediate addition of the NaOH solution. The mixture was allowed to stir for 5 minutes in an ice bath. MnO_2_ was added until gas evolution ceased. The combined solution was split into aliquots, centrifuged, and decanted to obtain the final ONOO^−^ solutions. The concentration was assessed by UV-vis spectroscopy using *I*_max_ = 302 nm with *e* = 1670 M^−1^ cm^−1^.

### Animal models of epilepsy

A chronic epilepsy model in male BALB/c mice (weighing 18 ± 2 g and aged 6 weeks) was developed following the guidelines established by Shimada and Yamagata.^37^ This literature-based procedure entailed administering daily injections of 100–150 μL of 10 mM PTZ in a sterile 0.9% w/v NaCl solution over five days, achieving a dosage range of about 50–75 mg kg^−1^. The successful induction of the model was verified on the eighth day with a repeat dose of PTZ, as evidenced by the typical seizure manifestations observed in the mice, including rearing, tonic seizures, and falling (Fig. S11†). Isoflurane anesthesia was applied to mitigate the risk of convulsive fatal events. All animal experiments were conducted in accordance with the China National Standard GB/T 42011-2022 (Guidance for Ethical Review of Laboratory Animal Welfare). The experimental protocols were reviewed and approved by the Animal Care and Use Committee of Shenzhen Institutes of Advanced Technology, Chinese Academy of Sciences (Approval No. SIAT-IACUC-210317-YGS-RYG-A1863).

### *In situ* linear array PAI protocol

Mice were secured in a stereotactic frame with anterior clamps and ear bars, ensuring head stability were anesthetized using 1.5–2% isoflurane in oxygen at a flow rate of 0.6–0.8 L min^−1^. A heating pad was used to maintain body temperature. In preparation for the imaging studies discussed in the main text, the scalp was removed to expose the skull, and a 2 mm drill bit, angled at 45°, was used to carefully penetrate the skull above the hippocampus, avoiding brain damage.

MMn (400 mM, 10 μL in saline) was loaded into a 1.0 mm OD glass microneedle syringe, automated by a Nanoject II microinjector pump (Drummond Scientific). The syringe was precisely positioned using the stereotactic frame and inserted 2 mm into the skull. MMn was injected at a rate of 1 μL min^−1^ (total volume: 3 μL). A two-minute absorption period was allowed before syringe removal and cleaning of the injection site.

Following MMn administration, mice were transferred to the PA imaging platform. Isoflurane was discontinued, and PTZ (50–75 mg kg^−1^, Sigma-Aldrich, United States) was administered intraperitoneally to induce seizures. PA images of the hippocampus (wavelengths: 725 nm and 800 nm; fluence: 20 mJ cm^−2^; frequency: 30 Hz) were captured before and after seizure onset using a linear-array PAI system. For each cross-section, 100 frames were acquired to ensure data consistency.

### *In vivo* linear array PAI protocol

Pre-PA imaging of the mouse brain was conducted at two emission wavelengths (725 nm and 800 nm) with a fluence of 20 mJ cm^−2^ and a frequency of 30 Hz. Under 1.5–2% isoflurane anesthesia with an oxygen flow rate of 0.6–0.8 L min^−1^, mice received a tail vein injection of MMn (1000 mM, 150 μL in saline). Anesthesia was then discontinued, allowing a return to normal levels of neuronal activity.

The hippocampal region was monitored using a linear-array PAI system over the course of 60 minutes, a time chosen to allow drug clearance from the bloodstream. PTZ was then administered intraperitoneally (50–75 mg kg^−1^, Sigma-Aldrich, United States) to induce seizures, with imaging continued for an additional 30 minutes to monitor seizure-related neurochemical changes. Subsequent re-anesthetization was performed to mitigate the effects of PTZ-induced seizures.

### *In vivo* circular-array PAI protocol

Mice were anesthetized with 1.5–2% isoflurane containing O^2^ at a flow rate of 0.6–0.8 L min^−1^. MMn (1000 mM, 150 μL in saline) was injected *via* the tail vein, followed by removal of anesthesia. After 40 minutes, the mice were anesthetized again and secured onto a head-mounted adapter, after which anesthesia was discontinued to allow for normal physiological activity. Sixty minutes post-MMn injection, PTZ (50–75 mg kg^−1^, Sigma-Aldrich, United States) was administered intraperitoneally to induce seizures.

As seizure activity progressed, neurochemical fluctuations were monitored using a circular-array PAI system (725 nm and 800 nm; laser frequency: 20 Hz; fluence: 20 mJ cm^−2^), equipped with a 10 MHz, 512-element circular-array ultrasound transducer. To ensure accurate detection of seizure-related neurochemical changes, the transducer was first positioned at the skull surface and then lowered by 2 mm to align the imaging plane with key deep-brain regions, including the hippocampus and motor cortex. With this setup, the system achieved an axial resolution of ∼80 μm and a lateral resolution of ∼120 μm, generating a cross-sectional brain image every 50 ms. This frame rate is well-suited for capturing seizure-related ONOO^−^ fluctuations, which typically evolve over hundreds of milliseconds to minutes. To ensure comprehensive tracking of these dynamic biochemical changes, imaging was conducted for 30 minutes. Following data acquisition, mice were re-anesthetized to mitigate PTZ-induced seizures.

### Statistical analysis software and methods

Data were derived from experiments conducted in triplicate and are presented as the SEM. This approach ensures robust statistical representation. All image and data analyses were performed using MATLAB (version 2017b). This software facilitated detailed and precise processing, aligning with rigorous analytical standards.

## Conclusions

This study represents the first real-time visualization and tracking of epileptic seizures and is made possible by the combined use of an ONOO^−^ specific sensing probe and advanced PAI. The sensing ability reported here holds promise for enhancing our understanding of epilepsy pathogenesis and paves the way for potential advances in epilepsy management and treatment.

## Data availability

All data needed to evaluate the conclusions in the paper are present in the paper and/or the ESI.†

## Author contributions

Conceptualization: YGR, ACS, JLS, CBL and ZHL; methodology: YGR, TC and ACS; investigation: JQC, CBL and RKG; visualization: TC, JTB and JFA; supervision: JLS, ACS and CBL; synthesis: CVC and ACS; writing—original draft: YGR, CVC, and ACS; writing—review & editing: JLS, CBL, and YGR.

## Conflicts of interest

There are no conflicts to declare.

## Supplementary Material

SC-016-D5SC00568J-s001
