# Supplementary material for: Real-time visualization of epileptic seizures using photoacoustic imaging with a peroxynitrite-responsive manganese(ii) texaphyrin
Source: Chem Sci. 2025 Mar 18;16(16):6862–71. doi: 10.1039/d5sc00568j (PMC11917445; doi:10.1039/d5sc00568j)
Supplement: SC-016-D5SC00568J-s001 [file SC-016-D5SC00568J-s001.pdf]

Supplementary Information (SI) for Chemical Science.
This journal is © The Royal Society of Chemistry 2025

# Supplementary Materials for

## Real-time Visualization of Epileptic Seizure using Peroxynitrite Responsive Manganese(II) Texaphyrin

Yaguang Ren,[a†] Calvin V. Chau,[b†] Tao Chen,[f, a†] Jingqin Chen,[a] Yu Hu,[c] Zhonghua Lu,[c] James T. Brewster,[b] Jonathan F. Arambula,[e] Rongkang Gao,[a] Adam C. Sedgwick,[d*] Jonathan L. Sessler,[b*] and Chengbo Liu[a*]

[a]*Research Center for Biomedical Optics and Molecular Imaging, Key Laboratory of Biomedical Imaging Science and System, Shenzhen Institute of Advanced Technology, Chinese Academy of Sciences, Shenzhen, 518055, China;*

[b]*Department of Chemistry, The University of Texas at Austin, Austin, Texas 78712-1224, United States;*

[c]*Guangdong Provincial Key Laboratory of Brain Connectome and Behavior, The Brain Cognition and Brain Disease Institute, Shenzhen Institutes of Advanced Technology, Chinese Academy of Sciences; Shenzhen-Hong Kong Institute of Brain Science, Shenzhen 518055, China;*

[d]*Department of Chemistry, King's College London, 7 Trinity Street, London, SE1 1DB, U.K.;*

[e]*Department of Chemistry, The University of Texas at Austin, Austin, Texas 78712-1224, United States; OncoTEX, Inc. 3800 N. Lamar Blvd. Austin, Texas 78756;*

[f]*School of Optics and Photonics, Beijing Institute of Technology, Beijing, 100089, China;*

**This file includes:**

Materials and Methods
Supplementary Text
Figs. S1 to S11

**Other Supplementary Materials for this manuscript include the following:**

Movies S1 to S3

## Materials and Methods

**Synthesis and Characterization of manganese(II) texaphyrin (MMn)~~MMn~~**

MMn was synthesized following established protocols *(25, 34)*. For purification, MMn was processed using reversed-phase tC18 solid-phase extraction (SPE) columns (Waters Sep-Pak, 10 g). The elution was performed with a gradient of acetonitrile (10% to 35%) in either a 0.1 M ammonium acetate or a 0.1% acetic acid aqueous solution. Its purity was confirmed through reverse-phase high-performance liquid chromatography (RP-HPLC), aligning with prior analyses *(25)*. RP-HPLC was conducted using a Thermo Scientific Dionex Ultimate 3000 HPLC system, equipped with a photodiode array (PDA) detector. Separation was achieved on a Syncronis C18 column (5 µm, 4.6 x 250 mm, Thermo Scientific). The mobile phase comprised a gradient of acetonitrile (10% to 99% over 20 minutes) in water, both containing 0.1% acetic acid, at a flow rate of 1.2 mL/min. MMn detection was conducted at wavelengths of 254, 470, and 740 nm. UV-vis spectra were obtained with a Varian Cary 5000 spectrophotometer at ambient temperature. A 10 mm path length quartz cuvette was used for all measurements.

***In vitro* Synthesis and Characterization of peroxynitrite ($ONOO^-$)~~$ONOO^-$~~**

Four separate solutions (10 mL) of $NaNO_2$ (0.6 M), HCl (0.68 M) with $H_2O_2$ (0.72 M), and NaOH (3.6 M) were prepared and stored in the freezer at -20 °C for 30 minutes. Then, the solutions were transferred to an ice bath and allowed to sit for 30 minutes. The $NaNO_2$ solution was added to a 100 mL beaker and placed in an ice bath with >500 rpm stirring. The $HCl/H_2O_2$ solution was added to the $NaNO_2$ solution, followed by immediate addition of the NaOH solution. The mixture was allowed to stir for 5 minutes in an ice bath. $MnO_2$ was added until gas evolution ceased. The combined solution was split into aliquots, centrifuged, and decanted to obtain the final $ONOO^-$ solutions. The concentration was assessed by UV-vis spectroscopy using $l_{max}$ = 302 nm with $\varepsilon$ = 1670 $M^{-1}cm^{-1}$.

**Animal Models of Epilepsy**

A chronic epilepsy model in male BALB/c mice (weighing 18 ± 2 g and aged 6 weeks) was developed following the guidelines established by Shimada and Yamagata *(35)*. This literature-based procedure entailed administering daily injections of 100-150 µL of 10 mM PTZ in a sterile 0.9% w/v NaCl solution over five days, achieving a dosage range of about 50-75 mg/kg. The successful induction of the model was verified on the eighth day with a repeat dose of PTZ, as evidenced by the typical seizure manifestations observed in the mice, including rearing, tonic seizures, and falling (Fig. S11). Isoflurane anesthesia was applied to mitigate the risk of convulsive fatal events.

***In situ* Linear Array PAI Protocol**

Mice were secured in a stereotactic frame with anterior clamps and ear bars, ensuring head stability were anesthetized using 1.5-2% isoflurane in oxygen at a flow rate of 0.6-0.8 L/min. A heating pad was used to maintain body temperature. In preparation for the imaging studies discussed in the main text, the scalp was removed to expose the skull, and a 2 mm drill bit, angled at 45°, was used to carefully penetrate the skull above the hippocampus, avoiding brain damage.

MMn (400 mM, 10 µL in saline) was loaded into a 1.0 mm OD glass microneedle syringe, automated by a Nanoject II microinjector pump (Drummond Scientific). The syringe was precisely positioned using the stereotactic frame and inserted 2 mm into the skull. MMn was injected at a rate of 1 µL/min (total volume: 3 µL). A two-minute absorption period was allowed before syringe removal and cleaning of the injection site.

Following MMn administration, mice were transferred to the PA imaging platform. Isoflurane was discontinued, and PTZ (50-75 mg/kg, Sigma-Aldrich, United States) was administered intraperitoneally to induce seizures. PA images of the hippocampus (wavelengths: 725 nm and 800 nm; fluence: 20 mJ/cm²; frequency: 30 Hz) were captured before and after seizure onset using a linear-array PAI system. For each cross-section, 100 frames were acquired to ensure data consistency.

***In vivo* Linear Array PAI Protocol**

Pre-PA imaging of the mouse brain was conducted at two emission wavelengths (725 nm and 800 nm) with a fluence of 20 mJ/cm² and a frequency of 30 Hz. Under 1.5-2% isoflurane anesthesia with an oxygen flow rate of 0.6-0.8 L/min, mice received a tail vein injection of MMn (1000 mM, 150 µL in saline). Anesthesia was then discontinued, allowing a return to normal levels of neuronal activity.

The hippocampal region was monitored using a linear-array PAI system over the course of 60 minutes, a time chosen to allow drug clearance from the bloodstream. PTZ was then administered intraperitoneally (50-75 mg/kg, Sigma-Aldrich, United States) to induce seizures, with imaging continued for an additional 30 minutes to monitor seizure-related neurochemical changes. Subsequent re-anesthetization was performed to mitigate the effects of PTZ-induced seizures.

***In vivo* Circular-Array PAI Protocol**

Mice were anesthetized with 1.5-2% isoflurane containing $O^2$ at a flow rate of 0.6-0.8 L/min. MMn (1000 mM, 150 µL in saline) was injected via the tail vein, followed by removal of anesthesia. After 40 minutes, the mice were anesthetized and

fixed onto a head-mounted adapter, after which anesthesia was removed. Once the mice were awake, intraperitoneal injection of PTZ (50-75 mg/kg, Sigma-Aldrich, United States) was performed 60 minutes after MMn injection. The activity in the cerebral cortex was monitored using a circular-array PAI system (725 nm and 800 nm; laser frequency: 20 Hz; fluence: 20 mJ/cm$^2$; central frequency of transducer: 10 MHz, 512 elements) for 30 minutes, and the mice were anesthetized again to alleviate drug-induced seizures.

**Statistical Analysis Software and Methods**

Data were derived from experiments conducted in triplicate and are presented as the SEM. This approach ensures robust statistical representation. All image and data analyses were performed using MATLAB (version 2017b). This software facilitated detailed and precise processing, aligning with rigorous analytical standards.

**Supplementary Text**

**Mechanism of Interaction Between Manganese(II) Texaphyrin (MMn) and Peroxynitrite ($ONOO^–$)**

Interactions between manganese(II) texaphyrin (MMn) and peroxynitrite ($ONOO^–$) were restudied and found to parallel what had been seen previously *(36)*. For instance, MMn was found to facilitate the decomposition of $ONOO^–$ to nitrogen dioxide and subsequently to the less reactive and relatively harmless species, nitrate and nitrite anions, in aqueous solutions via formation of an adduct to the Mn(II) center and homolysis of the O–O bond to form the oxidized Mn(III) texaphyrin. Reduction of the Mn(III)-texaphyrin back to the parent Mn(II) texaphyrin serves to complete the catalytic cycle and is promoted by reducing agents, such as ascorbate, glutathione, and hydrogen peroxide *(36)*.

**Utilizing Dual-Wavelength Photoacoustic Imaging (PAI) to Isolate MMn Signals from Blood Interference**

For the present study, we implemented a dual-wavelength photoacoustic imaging (PAI) strategy, tailored to differentiate the photoacoustic (PA) signals of MMn from those arising from blood. Key to this approach was the precise assessment of the fluence difference between two wavelengths. Before extraction of the MMn signal, we assessed the fluence difference between 725 nm and 800 nm to account for tissue absorption and scattering at depths of 2-3 mm within the brain. Calculation of the absorption and scattering coefficients for brain tissue at the respective wavelengths was based on data from a previous study *(37)*. The fluence variance between 725 nm and 800 nm at these specific depths was quantified as ranging from 2.0% to 5.8%, which is considered negligible, especially when compared to MMn absorption at 725 nm and 800 nm. Specifically, the minimal MMn absorption seen at 800 nm, rendered its PA signal at this wavelength effectively negligible. This allowed us to attribute the PA signal at 800 nm predominantly to blood. Utilizing the known absorption coefficients of blood at these two wavelengths (725 nm: 368.2 $cm^{-1}$ and 800 nm: 816 $cm^{-1}$), we could then infer the PA signal contribution of blood at 725 nm. A differential approach embodied in the equation below was used to extract the MMn PA signal at 725 nm.

$$\begin{cases} \Delta PA_{725Blood} = (\Delta PA_{800}/816) \times 368.2 \\ \Delta PA_{725MMn} = \Delta PA_{725} - \Delta PA_{725MMn} \end{cases}$$

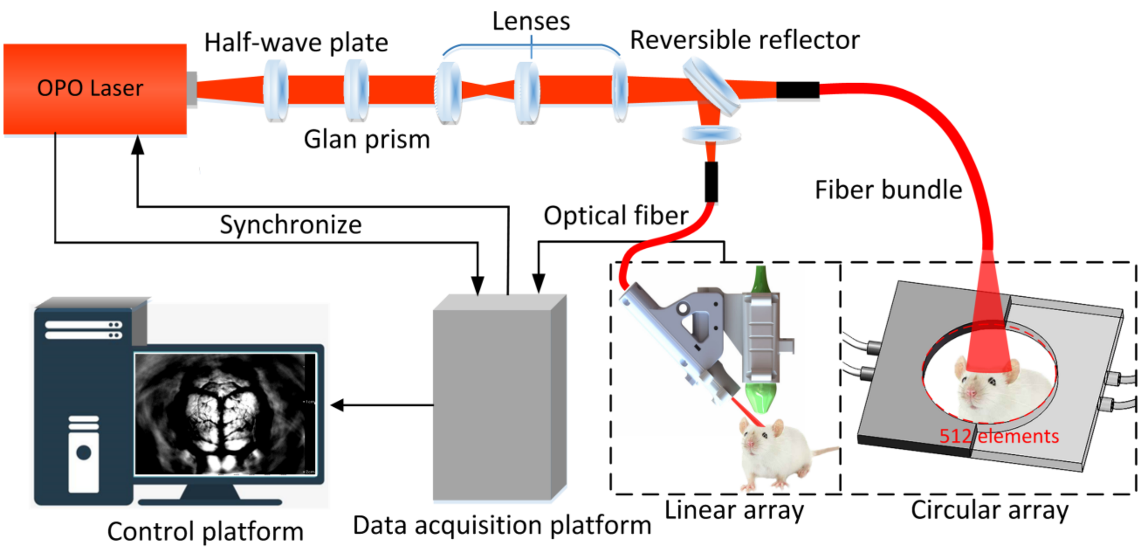


**Figure S1.** Scheme showing the linear array and circular array PAI system. An optical parametric oscillator (OPO) laser source (Innolas GmbH, Bonn, Germany) emitting pulsed lasers (8 ns) was coupled to a multi-mode optical fiber (1500 µm core diameter)/fiber bundles (diameter: 6.4 mm) for the PA signal excitation. The generation of ultrasonic waves produced from the brain were recorded by a customized 128-element linear array (center frequency: 15 MHz, bandwidth: > 60%, Vermon) for the linear-array PAI system and 521-element circular array ultrasound transducer (center frequency: 10 MHz, bandwidth: > 70%, Doppler) for the circular array PAI system. The Vantage 64LE (Verasonics, Inc., Kirkland, WA, USA) ultrasound system was used in the linear-array PAI system and Two 256-channel DAQ systems (MarsonicsDAQ256, Beijing TsingPai Technology Co., Ltd, China) synchronized with the excitation laser were used in Circular-Array PAI system. PA signals were acquired at a frame rate equal to the laser pulse firing frequency (30 Hz for the linear-array PAI system and 20 Hz for the circular array system). Conventional delay and sum reconstruction algorithm were used to reconstruct the PA images.

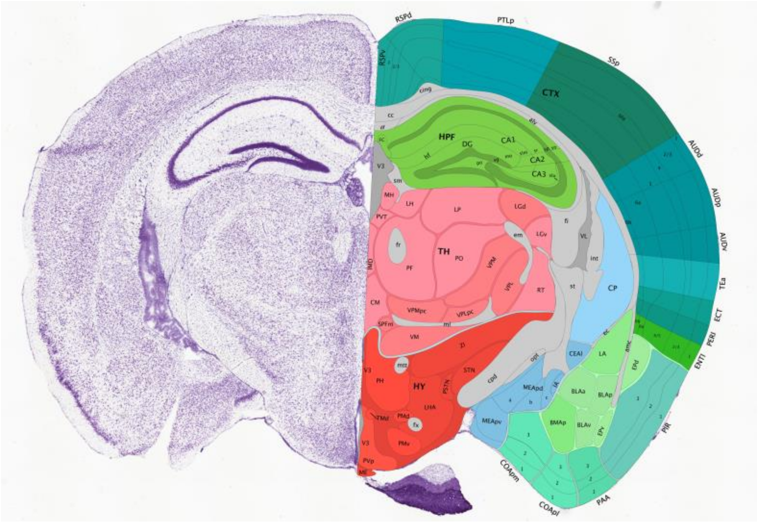


**Figure S2.** Schematic representation of the coronal plane in the mouse brain as the imaging plane for PAI using a linear-array system. The injection site, highlighted in green and labeled HPF, marks the hippocampal region. Credit: Allen Institute, Atlas brain maps: Interactive Atlas Viewer :: Atlas Viewer (brain-map.org).

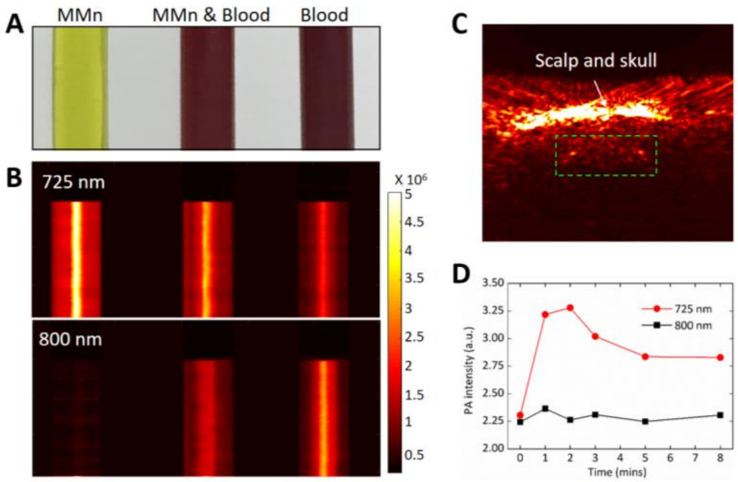


**Figure S3.** Experimental study of the feasibility of dual wavelength PAI for ONOO⁻ detection. (**A**) Photograph of the test solutions in plastic capillaries used in the PA studies. (**B**) PA signals of the samples in (**A**) under 725 nm and 800 nm photoexcitation, respectively. (**C**) PA image of the coronal plane of the mouse brain. (**D**) Mean value of the PA signals in the box marked in (**C**) at 725 nm and 800 nm over time.

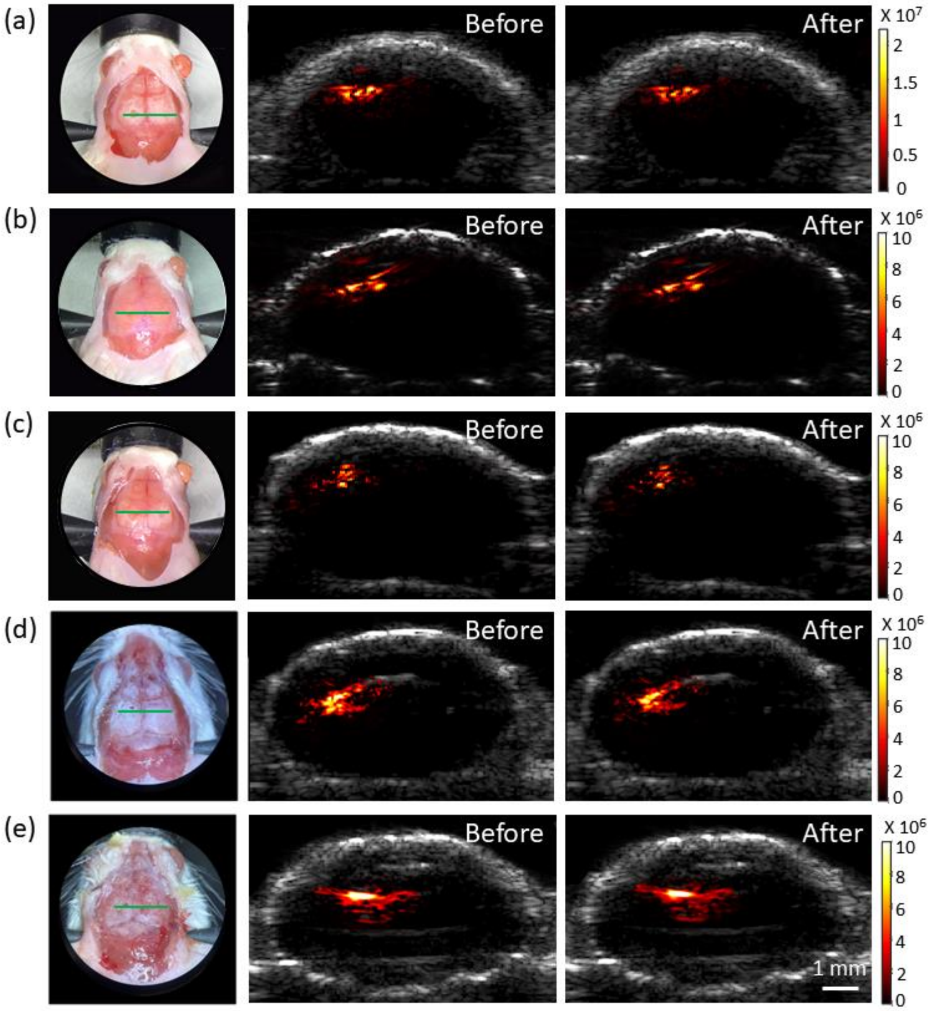


**Figure S4.** PAI studies of repeat epileptic mice subject to *in situ* cerebral injection of **MMn** before and after the PTZ-induced seizures. (**A**) Mouse 1: PA signal decrease of 15.0 ± 2.0% post PTZ-induced seizure. (**B**) Mouse 2: PA signal decrease of 8.0 ± 2.0% post PTZ-induced seizure. (**C**) Mouse 3: PA signal decrease of 19.4 ± 4.0% post PTZ-induced seizure.（D）Mouse 4: PA signal decrease of 16.1 ± 2.1% post PTZ-induced seizure. （E）Mouse 5: PA signal decrease of 18.1 ± 4.1% post PTZ-induced seizure. The deviation for each study was measured as the standard error of the mean (SEM).

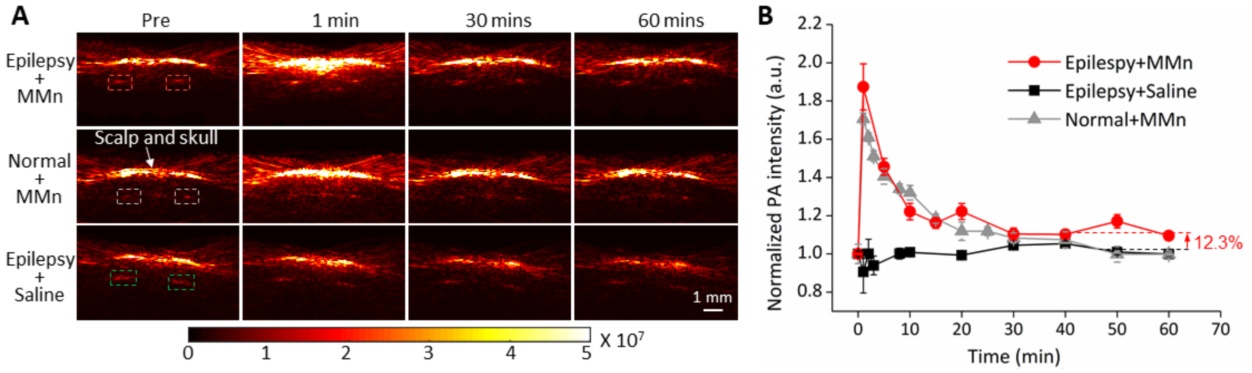


**Figure S5.** PAI of mouse brain (coronal plane) monitored over time. (**A**) PA image of the epileptic (top) and normal (middle) mouse brain before and after intravenous injection of **MMn** (1000 mM, 150 µL in saline). PA image of the epileptic mouse brain before and after intravenous injection of saline (lower) via tail vein. (**B**) Averaged PA intensity of the region in the hippocampus marked by the two green boxes.

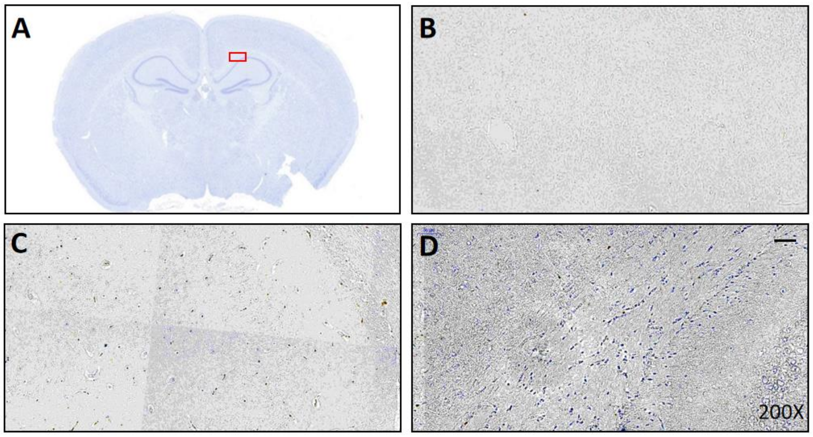


**Figure S6.** Scanning electron microscope images of brain slices. (**A**) Nissl staining of the brain slice shows the coronal plane of the mouse hippocampus site. (**B**) Scanning electron microscope image of normal mouse injected with MMn (1000 mM, 150 µL) via the tail vein in the position shown by the red rectangle in (**A**). Scanning electron microscope image of epileptic mouse injected with (**C**) MMn and (**D**) Evans blue in the same position. The scale bar (upper right of the image) corresponds to 50 µm.

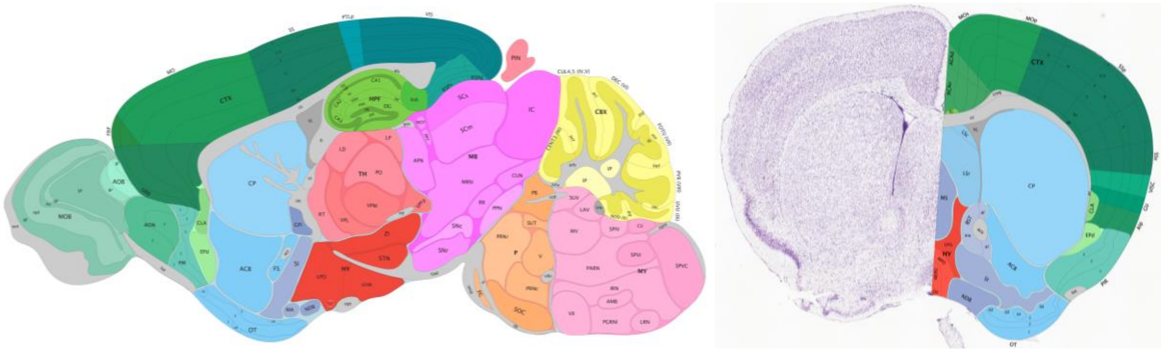


**Figure S7.** Schematic illustration of a sagittal view of a mouse brain (left), highlighting the hippocampal region in bright green and labeled HPF and the motor cortex region in dark green and labeled MO, identified as the areas of interest for photoacoustic imaging (PAI) using a circular-array system. Additionally, the corresponding coronal view of the motor cortex is shown on the right, with the motor areas labeled as MOs and MOp. Credit: Allen Institute, Atlas brain maps: Interactive Atlas Viewer :: Atlas Viewer (brain-map.org) (left); Interactive Atlas Viewer :: Atlas Viewer (brain-map.org) (right).

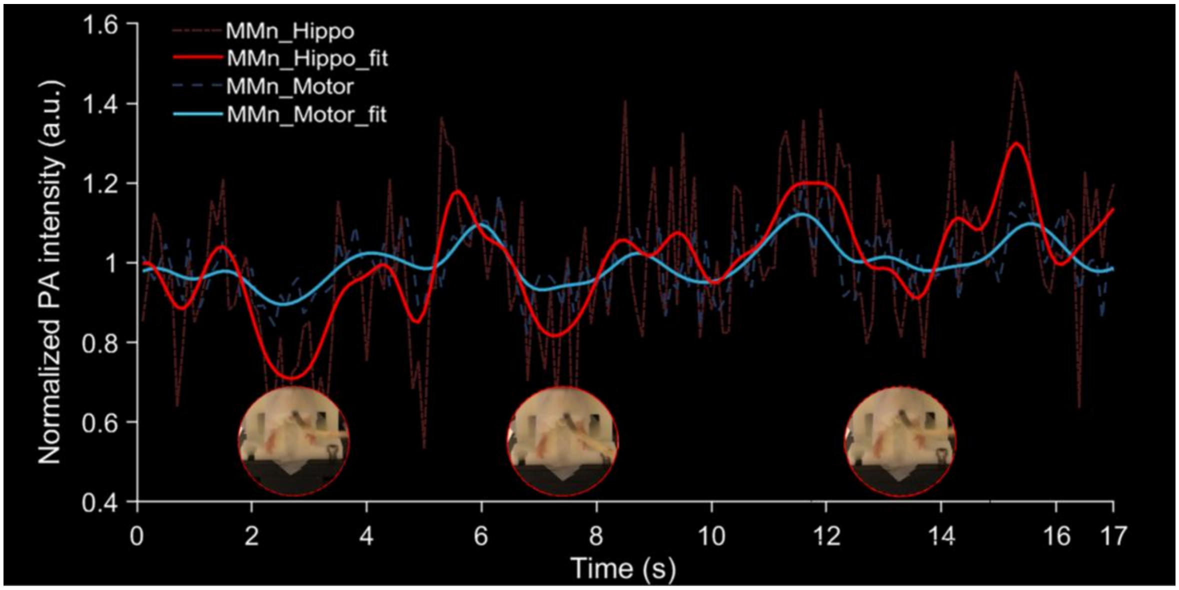


**Figure S8.** The averaged MMn signal in the hippocampal region (red dashed curve) and motor cortex region (blue dashed curve) over all time points from a 17-second segment of recorded data in Mouse #2. A Fast Fourier transform based low pass filter was used to analyze the changes in the averaged MMn signal. Results are shown for the red solid curve and blue solid curve, respectively. A cyclical decreasing trend in the MMn signal for both regions is noted; it is associated with the onset of an epileptic seizure as indicated by mouse tail curling.

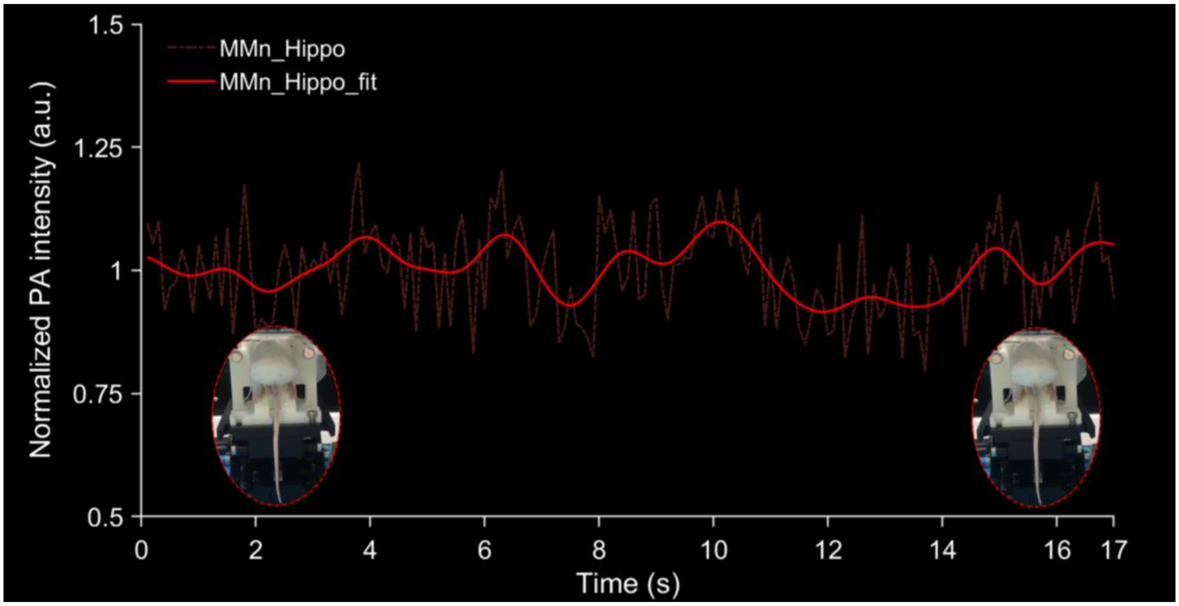


**Figure S9.** The averaged PA signal of MMn in the hippocampal region (red dashed curve) over all time points from a 17-second segment of recorded data before injection of PTZ to induce seizures. A Fast Fourier transform-based low-pass filter was used to analyze the changes of the averaged MMn signal and the results are shown using a red solid curve. No mouse tail curling nor obvious decreasing trend in the MMn signal was observed.

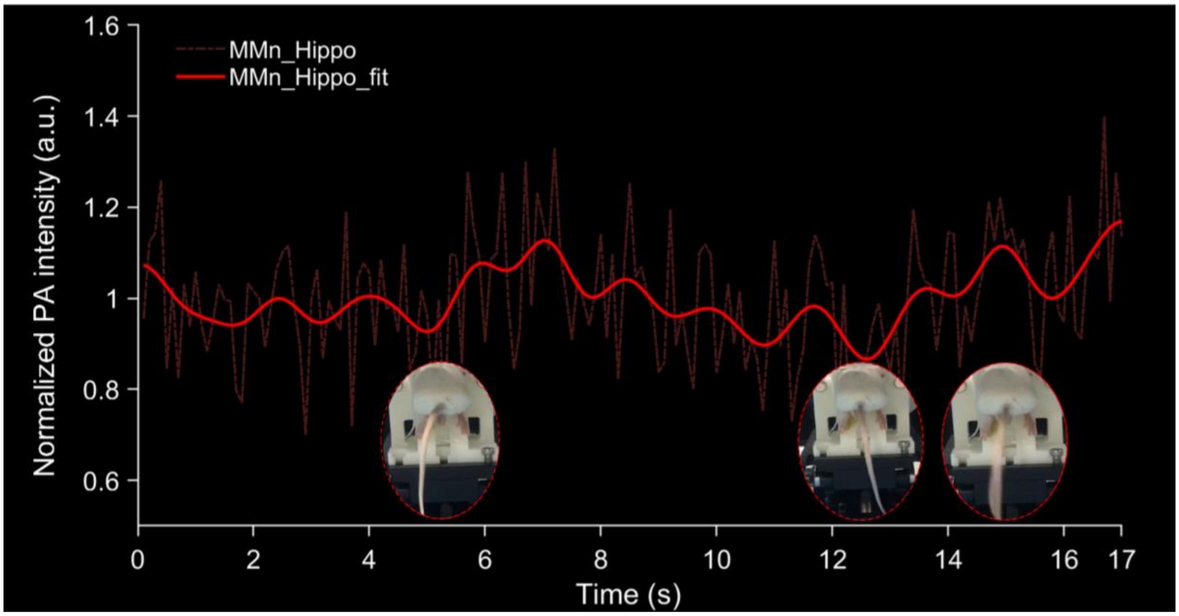


**Figure S10.** The averaged PA signal of MMn in the hippocampal region (red dashed curve) over all time points from a 17-second segment of recorded data after seizures had taken place for a while. A Fast Fourier transform-based low-pass filter was used to analyze the changes of the averaged MMn signal and the results are shown by the red solid curve. During PA monitoring, slight mouse tail curling and non-uniform cyclical changes in the MMn signal were noted.

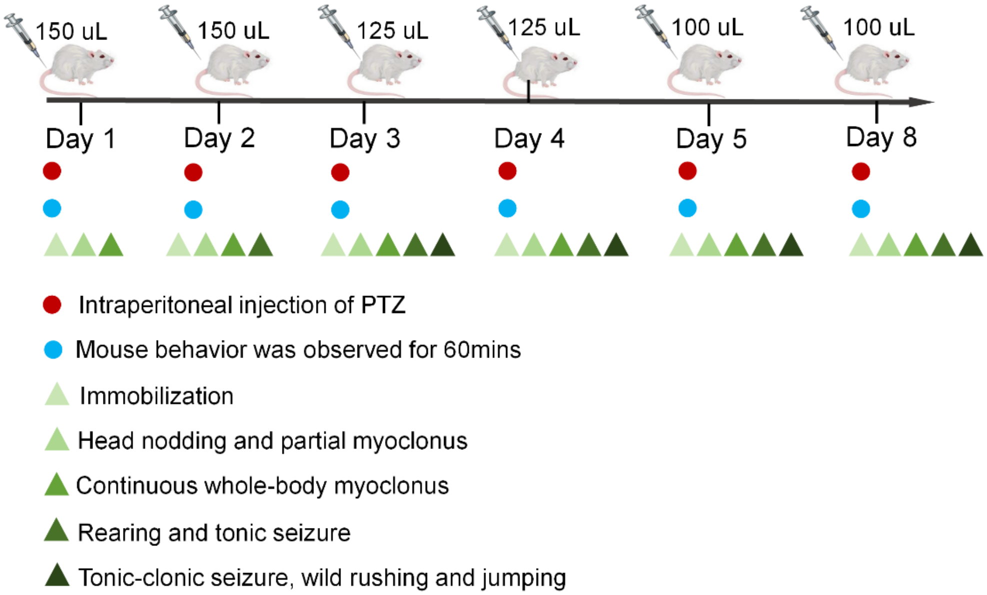


**Figure S11.** Regimen used to induce severe seizures in a PTZ-Kindling epilepsy mouse model. Daily injections of 100-150 µL of 10 mM PTZ in a sterile 0.9% w/v NaCl solution.

Seizure Levels:
Level 1: Immobilization;
Level 2: Head nodding and partial myoclonus;
Level 3: Continuous whole-body myoclonus;
Level 4: Rearing; tonic seizure;
Level 5: Tonic-clonic seizure, wild rushing and jumping.

**Reference**

25. J. T. Brewster, G. D. Thiabaud, P. Harvey, H. Zafar, J. F. Reuther, S. Dell'Acqua, R. M. Johnson, H. D. Root, P. Metola, A. Jasanoff, L. Casella, J. L. Sessler, Metallotexaphyrins as MRI-Active Catalytic Antioxidants for Neurodegenerative Disease: A Study on Alzheimer's Disease. *Chem.* **6**, 703-724 (2020).

34. Y. Ren, A. C. Sedgwick, J. Chen, G. Thiabaud, C. V. Chau, J. An, J. F. Arambula, X. He, J. S. Kim, J. L. Sessler, C. Liu, Manganese(II) Texaphyrin: A Paramagnetic Photoacoustic Contrast Agent Activated by Near-IR Light. *J. Am. Chem. Soc.* **142**, 16156-16160 (2020).

35. T. Shimada, K. Yamagata, Pentylenetetrazole-Induced Kindling Mouse Model. *J. Vis. Exp.* **136**, e56573 (2018).

36. R. Shimanovich, S. Hannah, V. Lynch, N. Gerasimchuk, T. D. Mody, D. Magda, J. Sessler, J. T. Groves, Mn(II)-Texaphyrin as a Catalyst for the Decomposition of Peroxynitrite. *J. Am. Chem. Soc.* **123**, 3613-3614 (2001).

37. S. L. Jacques, Optical properties of biological tissues: a review. *Phys. Med. Biol.* **58**, R37 (2013).
